# The Quick Physical Activity Rating (QPAR) scale: A brief assessment of physical activity in older adults with and without cognitive impairment

**DOI:** 10.1371/journal.pone.0241641

**Published:** 2020-10-30

**Authors:** James E. Galvin, Magdalena I. Tolea, Amie Rosenfeld, Stephanie Chrisphonte

**Affiliations:** Comprehensive Center for Brain Health, Department of Neurology, University of Miami Miller School of Medicine, Miami, Florida, United States of America; Nathan S Kline Institute, UNITED STATES

## Abstract

**Introduction:**

Alzheimer’s disease and related dementias (ADRD) currently affect over 5.7 million Americans and over 35 million people worldwide. At the same time, over 31 million older adults are physically inactive with impaired physical performance interfering with activities of daily living. Low physical activity is a risk factor for ADRD. We examined the utility of a new measure, the Quick Physical Activities Rating (QPAR) as an informant-rated instrument to quantify the dosage of physical activities in healthy controls, MCI and ADRD compared with Gold Standard assessments of objective measures of physical performance, fitness, and functionality.

**Methods:**

This study analyzed 390 consecutive patient-caregiver dyads who underwent a comprehensive evaluation including the Clinical Dementia Rating (CDR), mood, neuropsychological testing, caregiver ratings of patient behavior and function, and a comprehensive physical performance and gait assessment. The QPAR was completed prior to the office visit and was not considered in the clinical evaluation, physical performance assessment, staging or diagnosis of the patient. Psychometric properties including item variability and distribution, floor and ceiling effects, strength of association, known-groups performance, and internal consistency were determined.

**Results:**

The patients had a mean age of 75.3±9.2 years, 15.7±2.8 years of education and were 46.9% female. The patients had a mean CDR-SB of 4.8±4.7 and a mean MoCA score of 18.6±7.1 and covered a range of healthy controls (CDR 0 = 54), MCI or very mild dementia (CDR 0.5 = 161), mild dementia (CDR 1 = 92), moderate dementia (CDR 2 = 64), and severe dementia (CDR 3 = 29). The mean QPAR score was 20.2±18.9 (range 0–132) covering a wide range of physical activity. The QPAR internal consistency (Cronbach alpha) was very good at 0.747. The QPAR was correlated with measures of physical performance (dexterity, grip strength, gait, mobility), physical functionality rating scales, measures of activities of daily living and comorbidities, the UPDRS, and frailty ratings (all p < .001). The QPAR report of physical activities was able to discriminate between individuals with impaired physical functionality (32.2±23.9 vs 15.2±13.8, p < .001), falls risk (28.4±21.6 vs. 14.5±13.2, p < .001), and the presence of frailty (28.1±22.7 vs. 11.8±9.4, p < .001). The QPAR showed strong psychometric properties and excellent data quality, and worked equally well across different patient ages, sexes, informant relationships, and in individuals with and without cognitive impairment.

**Discussion:**

The QPAR is a brief detection tool that captures informant reports of physical activities and differentiates individuals with normal physical functionality from those individuals with impaired physical functionality. The QPAR correlated with Gold Standard assessments of strength and sarcopenia, activities of daily living, gait and mobility, fitness, health related quality of life, frailty, global physical performance, and provided good discrimination between states of physical functionality, falls risk, and frailty. The QPAR performed well in comparison to standardized scales of objective physical performance, but in a brief fashion that could facilitate its use in clinical care and research.

## Introduction

Alzheimer’s disease and related dementias (ADRD) currently affect over 5.7 million Americans and over 35 million people worldwide [1]. The number of ADRD cases is expected to increase 3-fold by the year 2050 as the number of older adults are increase [1–3]. In addition to cognitive impairment, over 31 million adults aged 50 and above are physically inactive [4] and impaired physical performance may interfere with activities of daily living (ADLs) [5]. Multiple lines of evidence indicate that decrements in physical health or frailty are risk factors for the development of cognitive impairment [6, 7] supported by both cross-sectional [8–10] and longitudinal analyses [11–13]. Impairment in physical performance may be a harbinger of future mild cognitive impairment (MCI) [14] and ADRD [13, 15] and may give clues to the presence of preclinical disease [16]. The cognitive-physical impairment relationship may have a bidirectional effect [11, 17], or may be influenced by the underlying etiology of cognitive impairment [18]. In addition to the direct relationship between cognitive and physical functionality, these two processes share may common risk factors, including cardiovascular, cerebrovascular, inflammatory, and metabolic derangements [6]. Of particular interest to ADRD prevention efforts, physical activity along with cognitive activity, social engagement, and diet are modifiable risk factors [19].

It is a challenge for clinicians and researchers to capture *physical activity* in an objective fashion. Objective measures of *physical performance and physical functionality* may be captured in several ways including analyses of gait and mobility, muscle mass and strength, global ratings of physical performance, frailty scales, or ratings of performance on ADLs. The ability to perform ADLs, particularly the more complex activities such as shopping, preparing meals, using appliances, and balancing a checkbook, rely a great deal on maintaining cognitive and physical function [11]. Direct measurement of physical activity is not as easily achieved. One option is to directly observe the patient, but this is not practical either for research or clinical care. A second option would be to have individuals wear sensors that capture steps and distance traveled or other forms of movement; however, this might be impractical in clinical settings or in large epidemiological studies and depending of the number of, and types of sensors worn might fail to capture arm movements, balance, and fine motor activities, or activities done in a seated position. A third option would be to capture ratings of physical activity through brief, validated patient-reported outcome measures (PROMs) [20–23]. PROMs may provide valid information about the patient’s physical activities and how they function on a daily basis. However, the reliability and validity of PROMs for physical activities in individuals with cognitive impairment has not been well established. A final option is to capture data describing physical activity from a reliable informant, similar to the administration of global rating scales such as the Clinical Dementia Rating [24] and the Quick Dementia Rating System [25].

Our hypothesis is that a physically active individual would have higher physical performance and functionality, and less impairments in ADLs and frailty compared with an individual with little regular physical activity. Given the facts that ADRD is a significant public health problem, physical activity is an important modifiable risk factor for ADRD, physical activity is related to physical functioning–particularly in people at risk of ADRD, and the challenges of measuring physical activity facing clinicians and researchers investigating ADRD, we created a new measure, the Quick Physical Activities Rating (QPAR). The QPAR is an informant-reported instrument that quantifies the “dosage” of physical activity in healthy controls, MCI and ADRD. We examined the performance of the QPAR with Gold Standard assessments of physical performance, physical functionality, and health including resting heart rate, manual dexterity, gait, mobility, strength, frailty, ADLs, health-related quality of life, and balance to establish the relationship between reported physical activity and physical functioning.

## Materials and methods

### Study participants

This study was conducted in 390 consecutive patient-caregiver dyads attending our center for clinical care or participation in cognitive aging research. During the visit, the patient and caregiver underwent a comprehensive evaluation including the Clinical Dementia Rating (CDR) and its sum of boxes (CDR-SB) [24], mood, neuropsychological testing, caregiver ratings of patient behavior and function, and a comprehensive physical performance and gait assessment. All components of the assessment are part of standard of care at our center [26]. This study was approved by the University of Miami Institutional Review Board.

### Development of the QPAR

**Fig 1:**
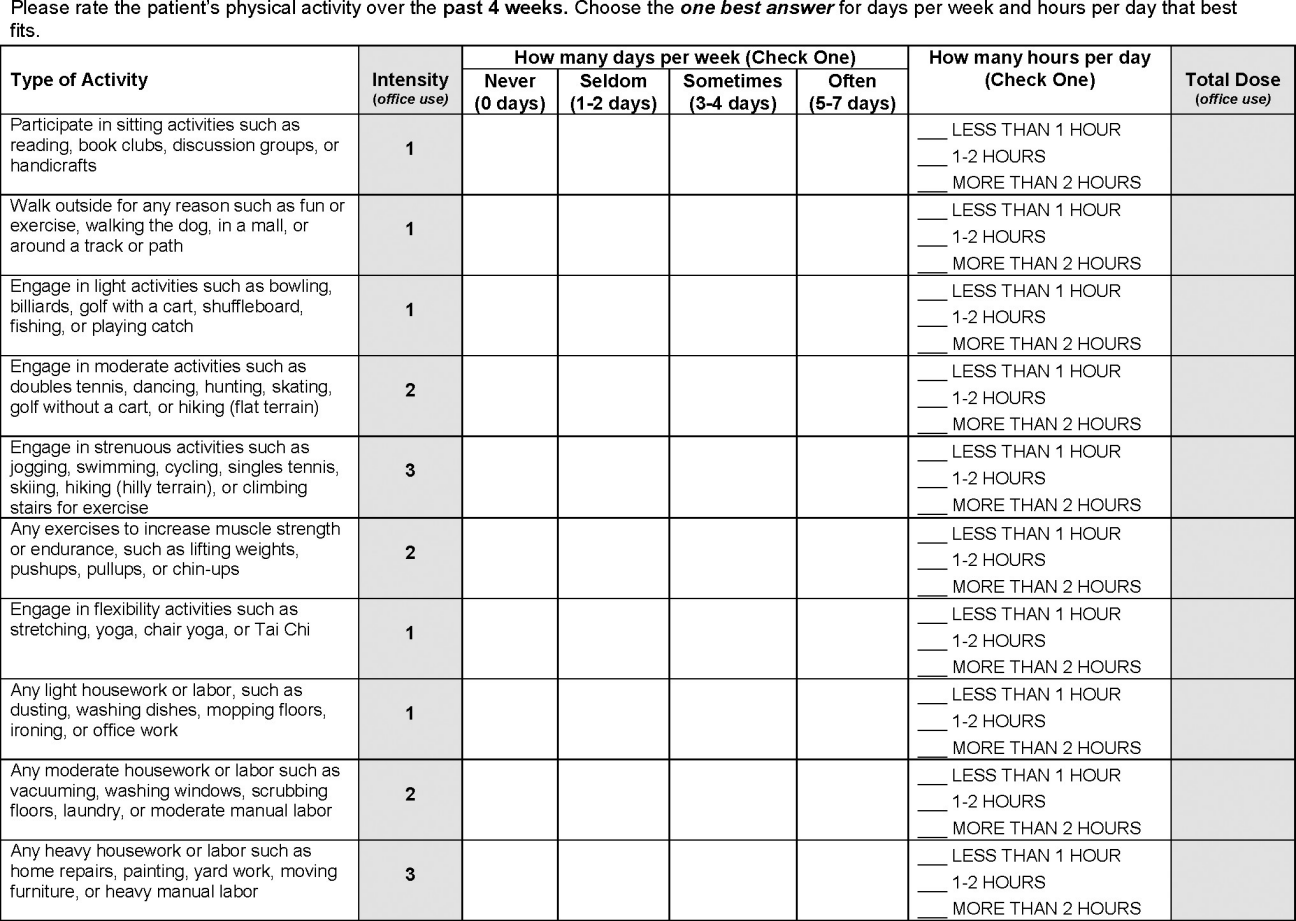
The Quick Physical Activity Rating (QPAR).

The QPAR (**Fig 1**) was developed as part of a review of a comprehensive assessment of older adults and their caregivers by a collaborative care team including a cognitive neurologist, gerontologist, physical therapist, nurse practitioners, and social workers. Items incorporated into the QPAR were captured as part of semi-structured interviews, rating scales, and physical and neurological performance measures. Final item selection was by consensus and included 10 items covering passive activities, walking, hobby and recreational activities, exercise, and housework with exemplars provided. Hobby activities, exercise and housework were further divided into light, moderate, and strenuous activities. Respondents were asked to consider these physical activities over the prior 4-week period. Each activity was weighted in intensity ranging from 1 (light) to 3 (heavy) intensity. Frequency of activity per week was collected as never (0 days), seldom (1–2 days), sometimes (3–4 days), and often (5–7 days). Duration of activity was collected as less than one hour per day, 1–2 hours per day, and more than two hours per day. Multiplication of the intensity (1–3), frequency (0–3) and duration (1–3) scores permitted calculation of a dose of physical activity ranging from 0–153. The QPAR took 3–5 minutes to complete.

### Administration of QPAR

Prior to the office visit, a welcome packet was mailed to the patient and caregiver to collect demographics and medical history and included the QPAR completed by the caregiver. The QPAR took 2–3 minutes to complete. The packets including the QPAR were returned prior to the appointment. The QPAR was not considered in the clinical evaluation, physical performance assessment, staging or diagnosis of the patient.

### Clinical assessment

The clinical assessments are modelled on the Uniform Data Set (UDS) 3.0 from the NIA Alzheimer Disease Center program [27, 28]. The CDR [24] was used to determine the presence or absence of dementia and to stage its severity; a global CDR 0 indicates no dementia; CDR 0.5 represents MCI or very mild dementia; CDR 1, 2, or 3 correspond to mild, moderate, or severe dementia. The CDR-SB was calculated by adding up the individual CDR categories giving a score from 0–18 with higher scores supporting more severe stages. Extrapyramidal features were assessed with the Movement Disorders Society-Unified Parkinson’s Disease Rating Scale, motor subscale part III (UPDRS) [29]. Activities of daily living were captured with the Functional Activities Questionnaire (FAQ) completed by the caregiver [30]. The Functional Comorbidity Index (FCI) [31] was used to measure overall health and medical comorbidities. The FCI contains 18 items rated as present or absent, with higher scores supporting greater co-morbidities that contribute to functional impairment. The Health Utilities Index-Mark 3 [32] was used to rate health-related quality of life and health status, which we have previously validated in dementia studies [33].

A 30-minute test battery was administered at the time of the office visit to assess the patient’s cognitive status. The psychometrician was unaware of the diagnosis, CDR stage or QPAR score. The Montreal Cognitive Assessment [34] was used for a global screen. The remaining components of the battery were modeled after the UDS battery used in the NIA Alzheimer Disease Centers [28] supplemented with additional measures: 15-item Multilingual Naming Test (naming) [28]; Animal naming and Letter fluency (verbal fluency) [28]; Hopkins Verbal Learning Task (episodic memory for word lists–immediate, delayed, and cued recall) [35]; Number forward/backward and Months backwards tests (working memory) [28]; Trailmaking A and B (processing and visuospatial abilities) [36]; and a novel Number-Symbol Coding Test (executive function). Mood was assessed with the Hospital Anxiety Depression Scale [37] providing subscale scores for depression (HADS-D) and anxiety (HADS-A). Diagnoses were determined using standard criteria for MCI [38], AD [39], Dementia with Lewy bodies (DLB) [40], vascular dementia (VaD) [41], and frontotemporal degeneration (FTD) [42].

### Physical performance assessment

A comprehensive assessment of manual dexterity gait, mobility, strength, frailty, and global physical performance was performed to test the hypothesis that higher reported physical activity would correspond to better physical performance. Clinicians were unaware of diagnosis, CDR stage or QPAR score. Anthropometric measurements of lean skeletal muscle and percent body fat were performed by bioimpedance using the InBody 770 (InBody Co, LTD, Cerritos, CA). Body mass index (BMI) was calculated from height and weight and abdominal-hip ratio was calculated by manual measurements. As a measure of fitness, resting pulse (sitting and standing) was recorded. Handgrip strength was measured with a handheld dynamometer (Baseline Digital Smedley Spring Dynamometer; Patterson Medical, Warrenville, IL) in each hand and expressed in kilograms (kg) and mean grip strength was calculated. Sarcopenia was measured using the Short Portable Sarcopenic Measure (SPSM) [43], used in our prior studies [8–10] and validated in older adult populations. The SPSM contains three measurements: lean BMI, grip strength by height, and 5 complete chair-raises, and is scored 0–18 with lower scores suggesting greater sarcopenia.

Upper extremity physical performance was measured with the Purdue Pegboard (PBB) [44]. The PBB is a test of coordination and fine fingertip dexterity testing the ability to manipulate small pegs into holes within 30 seconds with scores obtained for right hand, left hand, both hands, and right+left+both hands. A second task generates an assembly score with the participant manipulating pins, collars, and washers within 60 seconds.

Gait and mobility were assessed using the Timed Up and Go (TUG) task and computerized gait assessments. The TUG measures the time required for an individual to arise from a chair, walk 3m, turn around, walk back to the chair, and sit down [45, 46]. TUG times greater than 12–13.5 sec in community-dwelling adults are associated with increased falls risks [47, 48]. Gait characteristics were measured using a computerized walkway consisting of a pressure sensitive mat with a size of 20 ft. long x 4 ft. wide and gait analysis software. For the first 100 subjects GAITRite system (CIR Systems, PA) was used and for the remaining 170 subjects, a Zenomat system (ProtoKinetics LLC) was used. Previous studies have shown that the two systems have minimal differences [49]. For this study, gait velocity (m/sec) is used in analyses.

Global physical performance was captured with two similar scales: the mini Physical Performance Test (mPPT) [50] and the Short Physical Performance Battery (SPPB) [51]. The mPPT was used as the primary objective measure of overall physical performance in this study. The mPPT includes the following tasks: pick-up-penny, the 50-feet usual-pace walking test, 5 complete chair-raises, and the progressive Romberg balance test, each ranging from 0 to 4, with 4 indicating the highest level of performance (possible range of scores 0–16). A score of <12 was used as an indicator of impaired physical functionality [50]. The SPPB contains similar tasks as the mPPT except for the pick-up-penny task and is scored from 0–12 with lower scores implying greater functional impairment [51].

Physical frailty was assessed with the Fried Frailty Phenotype [52] and the Canadian Health and Aging (CHSA) Clinical Frailty Scale [53]. The Fried Scale is a five-factor frailty index which includes muscle weakness, slow gait, fatigue, physical inactivity, and weight loss. Scores of 1–2 are rated as pre-frailty and scores of 3 or greater support the presence of frailty [52]. The CHSA is a semi-quantitative global ordinal rating scale from 1–7. Scores of 5–7 signify mild, moderate, and severe frailty [53].

### Statistical analyses

Analyses were conducted with IBM SPSS Statistics v26 (Armonk, NY). Descriptive statistics were used to examine patient and caregiver demographic characteristics, informant rating scales, dementia staging, and physical performance testing. One-way analysis of variance (ANOVA) with Tukey-Kramer post-hoc tests were used for continuous data and Chi-square analyses were used for categorical data. To assess item variability, the item frequency distributions, range, and standard deviations were calculated. Kurtosis and skewness statistics were examined to characterize the shape and symmetry of the distribution. Kurtosis is a measure of the extent to which there are outliers. For a normal distribution, the value of the kurtosis statistic is zero. Positive kurtosis indicates that the data exhibit more extreme outliers than a normal distribution. Negative kurtosis indicates that the data exhibit fewer extreme outliers than a normal distribution. Skewness is a measure of the asymmetry of a distribution. The normal distribution is symmetric and has a skewness value of 0. A distribution with a significant positive skewness has a long right tail. A distribution with a significant negative skewness has a long left tail. As a guideline, a skewness value more than twice its standard error is taken to indicate a departure from symmetry. The QPAR was examined for floor and ceiling effects. Total QPAR scores and individual items were examined for their psychometric properties and compared with patient characteristics, rating scales, and physical performance.

Construct validity was examined based on the unified framework of construct validity [54, 55] examining six aspects: consequential (are there risks with invalid scores), content (does the test measure constructs of interest), substantive (is the theoretical foundation sound), structural (do interrelationships of test measurements correlated with construct of interest), external (does the test have convergent, discriminant, and predictive qualities), and generalizability (does the test work across different groups and settings). Strength of association was assessed comparing QPAR scores measuring dosage of physical activity with the mean performance on each Gold Standard measure of physical performance and functionality using Pearson correlation coefficients. We then compared individual QPAR items to Gold Standard measurements of cognition, function, physical performance, and aging using Spearman correlation coefficients. Known-group validity was assessed by examining the QPAR scores by patient and caregiver characteristics, CDR staging, and dementia etiology [25, 56]. Internal consistency was examined as the proportion of the variability in the responses that is the result of differences in the respondents, reported as the Cronbach alpha reliability coefficient. Coefficients greater than 0.7 are good measures of internal consistency [25, 56]. Receiver operator characteristic (ROC) curves were used to assess discrimination between patient functionality (mPPT), falls risk (TUG), and frailty (CHSA) and the QPAR. Results are reported as area under the curve (AUC) with 95% confidence intervals (CIs). Correction for multiple comparisons was performed using Bonferroni corrections.

## Results

### Sample characteristics

The patients had a mean age of 75.3±9.2 years, 15.7±2.8 years of education and were 46.8% female (**Table 1**). The caregivers had a mean age of 56.3±15.3 years, 16.0±2.6 years of education, and were 65.4% female. The sample was largely White (97%) and 6.4% reported Hispanic ethnicity. The patients had a mean CDR-SB of 4.8±4.7 and a mean MoCA score of 18.6±7.1. The mean Health Utilities Index-Mark 3 score was 0.518±0.3 suggesting moderate health-related quality of life. The mean QPAR score was 20.2±18.9 (range 0–132) covering a wide range of physical activity. This sample covered a range of healthy controls (CDR 0 = 54), MCI or very mild dementia (CDR 0.5 = 161), mild dementia (CDR 1 = 92), moderate dementia (CDR 2 = 64), and severe dementia (CDR 3 = 29). Caregivers were mostly spouses (69.4%), adult children (17.6%), or other individuals (13.0%) with 70.1% reporting living with the patient and 85.8% having daily contact.

**Table 1:** Sample characteristics (n = 390).

Patient Characteristics		Caregiver Characteristics	
Age, y	75.3 (9.2)	Age, y	56.3 (15.3)
Sex, %F	46.1	Sex, %F	62.7
Education, y	15.7 (2.8)	Education, y	16.0 (2.6)
Race, %White	97.4	Race, %White	92.9
Ethnicity, % Hispanic	6.4	Ethnicity, %Hispanic	8.0
CDR-SB	4.8 (4.7)	Relationship	
MoCA	18.6 (7.1)	%Spouse	69.4
FAQ	9.6 (9.8)	%Adult Child	17.6
Health Utilities Index	0.518 (0.3)	%Other	13.0
Fried Frailty Score	2.4 (1.4)	Lives with Patient, %Yes	70.1
mPPT	9.9 (3.5)	Sees Patient Daily, %Yes	85.8
UPDRS	10.4 (13.7)		
QPAR	20.2 (18.9)		

**Key:** CDR-SB = Clinical Dementia Rating Sum of Boxes; MoCA = Montreal Cognitive Assessment; FAQ = Functional Activities Questionnaire; mPPT = Mini Physical Performance Test; UPDRS = United Parkinson’s Disease Rating Scale; QPAR = Quick Physical Activity Rating.

### QPAR data quality

**Table 2** demonstrates the item distribution and inter-item correlation for the QPAR. The standard deviation was similar for all items, ranging from 1.4 to 6.0. The individual QPAR items were weakly correlated with each other suggesting that each question covered a different form of activity, however each item correlated moderately-to-strongly with the overall QPAR score. The degree to which the patient QPAR was free from random error was assessed by its internal consistency with Cronbach alpha (**Table 3**). The internal consistency was very good at 0.747. The QPAR covered nearly the entire range of possible scores and the mean, median and standard deviation demonstrated a sufficient dispersion of scores for assessing physical activity with a low percentage of missing data. The distribution statistics of the QPAR demonstrate a long right-sided tail with outliers encompassing individuals who report very high physical activity. There were low floor (4.4%) and ceiling (0%) effects. Thus, data quality for the QPAR were very good to excellent.

**Table 2:** QPAR item distributions, inter-item, and item-total correlations.

QPAR Item	Mean (SD)	Inter-Item Correlations										Item-Total R
		Q1	Q2	Q3	Q4	Q5	Q6	Q7	Q8	Q9	Q10	
Sitting activities (Q1)	3.3 (3.5)	1										.409
Walking (Q2)	2.6 (2.5)	.111	1									.510
Light Activities (Q3)	0.9 (1.4)	.180	.298	1								.541
Moderate Activities (Q4)	1.3 (3.3)	.145	.281	.474	1							.562
Strenuous Activities (Q5)	3.0 (6.0)	.201	.321	.335	.352	1						.735
Strength/Endurance Exercises (Q6)	2.0 (3.5)	.162	.253	.249	.276	.460	1					.552
Flexibility Exercises (Q7)	0.8 (1.6)	.167	.206	.237	.185	.340	.486	1				.512
Light Housework (Q8)	2.5 (2.7)	.218	.227	.244	.138	.281	.074	.236	1			.597
Moderate Housework (Q9)	3.1 (4.4)	.195	.247	.215	.139	.269	.150	.290	.699	1		.652
Heavy Housework (Q10)	1.8 (4.8)	.041	.222	.257	.327	.295	.129	.198	.369	.450	1	.622

**Table 3:** QPAR scale score features, distribution, and internal-consistency reliability statistics.

Scale	Items	Reliability	Score Features and Distribution									
		Cronbach alpha (95% CI)	Range	Mean	Median	SD	Skewness	SE	Kurtosis	SE	% Floor	% Ceiling
QPAR	10	.747 (.70-.79)	0–132	20.2	15.0	18.9	2.0	0.1	6.0	0.2	4.4	0.0

**Note:** Possible range of QPAR scores 0–153.

% Floor is the percentage who reported the lowest (worst) possible score.

% Ceiling is the percentage who reported the highest (best) possible score.

### Relationship of QPAR scores to physical performance

The strength of association between the physical activity reported by the QPAR and objective measures of physical performance, physical functionality, and anthropometric measurements is demonstrated in **Table 4**. The QPAR was showed moderate correlations with physical rating scales, gait measures, grip strength, gross motor and manual dexterity, measures of activities of daily living and comorbidities, the UPDRS, and frailty ratings (all p < .001). QPAR scores were inversely related to a measure of cardiovascular fitness, resting sitting (p = .004) and standing (p = .003) pulse rate and % Body Fat (p < .001). The QPAR had weaker associations with lean muscle mass and abdominal/hip ratio and was not associated with BMI.

**Table 4:** Concurrent validity with QPAR.

Variable	R	P-Value
mPPT	.458	**< .001**
SPPB	.444	**< .001**
SPSM	.365	**< .001**
TUG	-.278	**< .001**
Mean Grip Strength	.272	**< .001**
PPB Right+Left+Both	.321	**< .001**
PPB Assembly	.405	**< .001**
Gait Velocity	.402	**< .001**
Lean Skeletal Muscle	.131	.02
%Body Fat	-.218	**< .001**
BMI	.034	.59
Abdomen/Hip Ratio	-.155	.01
Resting Pulse, sitting	-.179	**.004**
Resting Pulse, standing	-.192	**.003**
FAQ	-.434	**< .001**
UPDRS	-.263	**< .001**
Health Utilities Index	.464	**< .001**
Fried Frailty Scale	-.456	**< .001**
CHSA	-.459	**< .001**
FCI	-.270	**.001**

**KEY:** mPPT = Mini Physical Performance Test; SPPB = Short Physical Performance Battery; SPSM = Short Portable Sarcopenia Measure; TUG = Timed Up and Go; PPB = Purdue Pegboard; BMI = Body Mass Index; FAQ = Functional Activities Questionnaire; UPDRS = Unified Parkinson’s Disease Rating Scale; CHSA = Canadian Health and Aging Clinical Frailty Scale; FCI = Functional Comorbidity Index.

**Bold** p-values signify significance after correction for multiple comparisons.

### Strength of association of the QPAR items

Strength of association of the QPAR is demonstrated in **Table 5** comparing individual QPAR items to Gold Standard measurements of cognition, function, physical performance, physical functionality, and frailty. After correction for multiple comparisons, there was convergence between QPAR items Walking (Q2), Strenuous Activities (Q4), Light (Q8), Moderate (Q9) and Heavy (Q10) Housework and age. All QPAR items except for Walking (Q2), Strength/Endurance (Q6) and Flexibility (Q7) were associated with CDR, FAQ, and MoCA scores. Strength/Endurance (Q6) was associated only with SPSM while Flexibility (Q7) was not associated with any measures. After correction, no QPAR items were associated with medical co-morbidities captured by the FCI.

**Table 5:** Strength of association of the QPAR items with outcome measures.

QPAR Item	Age	CDR	FAQ	FCI	MoCA	mPPT	SPPB	SPSM	PPB-Assembly	TUG	UPDRS	Fried
Sitting activities (Q1)	-.086 (.169)	**-.305 (< .001)**	**-.395 (< .001)**	-.148 (.078)	**.340 (< .001)**	**.227 (< .001)**	**.217 (.001)**	.064 (.322)	.219 (.021)	-.096 (.167)	**-.226 (< .001)**	**-.251 (< .001)**
Walking (Q2)	**-.247 (< .001)**	-.167 (.008)	-.176 (.005)	-.184 (.028)	.161 (.010)	**.322 (< .001)**	**.311 (< .001)**	**.239 (< .001)**	.120 (.210)	**-.224 (.001)**	-.112 (.088)	**-.236 (< .001)**
Light Activities (Q3)	-.128 (.040)	**-.246 (< .001)**	**-.258 (< .001)**	-.200 (.017)	**.282 (< .001)**	**.311 (< .001)**	**.315 (< .001)**	**.323 (< .001)**	.219 (.022)	**-.261 (< .001)**	-.110 (.093)	**-.351 (< .001)**
Moderate Activities (Q4)	-.157 (.012)	**-.197 (.002)**	**-.223 (< .001)**	-.211 (.012)	**.213 (.001)**	**.305 (< .001)**	**.315 (< .001)**	**.285 (< .001)**	.260 (.006)	**-.221 (.001)**	-.184 (.005)	**-.345 (< .001)**
Strenuous Activities (Q5)	**-.252 (< .001)**	**-.251 (< .001)**	**-.261 (< .001)**	-.157 (.061)	**.243 (< .001)**	**.334 (< .001)**	**.328 (< .001)**	**.352 (< .001)**	**.372 (< .001)**	-.122 (.079)	-.121 (.079)	**-.390 (< .001)**
Strength/Endurance (Q6)	-.086 (.169)	-.145 (.021)	-.127 (.044)	-.126 (.135)	.120 (.056)	.164 (.009)	.172 (.007)	**.249 (< .001)**	.195 (.041)	.016 (.821)	-.117 (.075)	-.198 (.006)
Flexibility Exercises (Q7)	-.047 (.459)	-.112 (.074)	-.122 (.052)	-.225 (.007)	.176 (.005)	.118 (.062)	.093 (.143)	.071 (.271)	**.287 (.002)**	-.084 (.223)	-.031 (.634)	-.107 (.097)
Light Housework (Q8)	**-.304 (< .001)**	**-.391 (< .001)**	**-.445 (< .001)**	-.114 (.176)	**.378 (< .001)**	**.402 (< .001)**	**.403 (< .001)**	.171 (.008)	.216 (.024)	**-.304 (< .001)**	**-.268 (< .001)**	**-.348 (< .001)**
Moderate Housework (Q9)	**-.256 (< .001)**	**-.299 (< .001)**	**-.316 (< .001)**	-.167 (.047)	**.243 (.002)**	**.337 (< .001)**	**.316 (< .001)**	.129 (.046)	.158 (.098)	**-.267 (< .001)**	**-.210 (.001)**	**-.272 (< .001)**
Heavy Housework (Q10)	**-.206 (.001)**	**-.232 (< .001)**	**-.205 (.001)**	-.113 (.180)	**.196 (.002)**	**.310 (< .001)**	**.300 (< .001)**	**.342 (< .001)**	.186 (.052)	**-.210 (.002)**	-.183 (.005)	**.272 (< .001)**

**NOTE: ρ**-coefficient (p-value); Bold represents significance after correction for multiple comparisons (corrected p < .004).

**KEY:** QPAR = Quick Physical Activity Rating; CDR = Clinical Dementia Rating; MoCA = Montreal Cognitive Assessment; FAQ = Functional Activities Questionnaire; FCI = Functional Comorbidity Index; mPPT = Mini Physical Performance Test; SPPB = Short Physical Performance Battery; SPSM = Short Portable Sarcopenia Measure; PPB = Purdue Pegboard; TUG = Timed Up and Go; UPDRS = United Parkinson’s Disease Rating Scale.

### Discriminability of the QPAR

**Fig 2:**
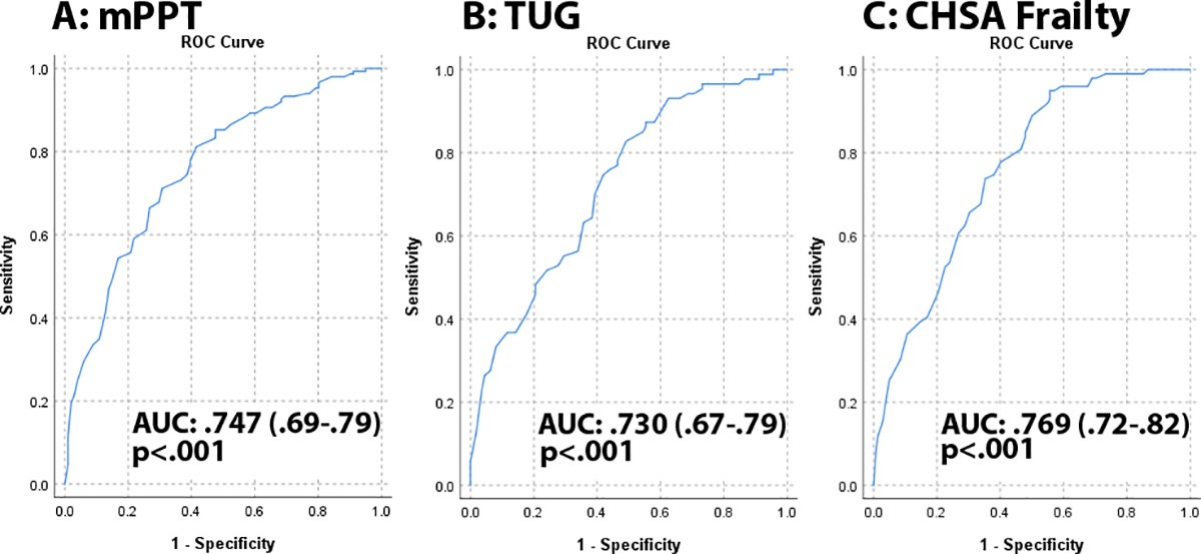
Receiver operator characteristics curve for QPAR. We tested the ability of the QPAR to discriminate between individuals with impaired physical functionality (mPPT), falls risk (TUG) and the presence of frailty (CHSA rating). Panel A: The QPAR demonstrated good discrimination between normal and impaired physical functionality as determined by mPPT scores with an AUC 0.747 (95%CI: 0.69–0.79, p < .001). Panel B: The QPAR showed good discrimination between low and increased falls risk determined by TUG times with an AUC 0.730 (95% CI: 0.67–0.79, p < .001). Panel C: The QPAR demonstrated good discrimination between individuals with and without frailty by CHSA staging with an AUC 0.769 (95% CI: 0.72–0.82, p < .001).

As our hypothesis was that individuals who reported higher physical activities would have better physical performance and functionality, we then tested the ability of the QPAR report of physical activities to discriminate between individuals with impaired physical functionality (mPPT), falls risk (TUG) and the presence of frailty (CHSA rating). The QPAR scores were significantly different between individuals with normal physical functionality and those with impaired functionality (28.5±22.0 vs 13.9±13.4, p < .001). The QPAR scores were significantly different between individuals with low falls risk and those with increased falls risk (24.9±21.0 vs. 11.4±10.0, p < .001). The QPAR scores were significantly different between non-frail individuals and those with frailty (26.3±21.0 vs. 10.9±9.9, p < .001). Individuals with impaired functionality, higher falls risk, and worse frailty ratings were older, had worse cognitive performance, and had poorer global physical performance across all measures (all p- < .001). The QPAR demonstrated good discrimination (**Fig 2**) between normal and impaired physical functionality as determined by mPPT scores with an AUC 0.747 (95%CI: 0.69–0.79, p < .001), between low and increased falls risk determined by TUG times with an AUC 0.730 (95% CI: 0.67–0.79, p < .001), and between individuals with and without frailty by CHSA staging with an AUC 0.769 (95% CI: 0.72–0.82, p < .001).

### Known-groups performance of QPAR

The performance of the QPAR as a report of physical activity and the mPPT as objective measure of physical functionality were compared between patient sex, race, ethnicity, caregiver relationship, age, CDR stages, and dementia etiologies in **Table 6**. There was no difference in mean QPAR scores for male or female patients or by the caregivers who completed the QPAR. African American patients may have more physical activity than White or Hispanic patients, but given the low number of non-White patients, the results should be interpreted with caution. Individual over 80 years old had less physical activity than other age groups. Both QPAR and mPPT scores declined with worsening cognitive ratings by CDR. Post-hoc analyses for QPAR revealed that CDR 0 patients were different from all other CDR stages. In individuals who were rated CDR 0.5 or higher, QPAR scores did not differ between adjacent CDR stages. QPAR scores in healthy controls were significantly different than MCI and all dementia etiologies, while MCI individuals were different from all dementia etiologies. QPAR scores were not different between dementia etiologies.

**Table 6:** QPAR and mPPT scores by sociodemographic characteristics, CDR staging, and dementia etiology.

	Sex			Race/ethnicity				Caregiver Relationship			
Scale	Male	Female	p-value	White	Black	Hispanic	p-value	Spouse	Child	Other	p-value
QPAR	19.2 (18.7)	21.2 (19.0)	0.29	19.6 (18.1)	37.1 (41.3)	21.9 (15.9)	0.02	20.2 (17.2)	19.1 (23.0)	20.8 (22.0)	0.88
mPPT	9.4 (3.5)	10.1 (3.5)	0.13	10.8 (3.5)	11.7 (3.1)	11.3 (2.5)	0.75	9.8 (3.4)	9.5 (3.9)	10.1 (3.9)	0.86
**Scale**	**<60y**		**60-69y**		**70-79y**		**80+y**		**p-value**		
QPAR	30.3 (18.3)		27.8 (22.9)		21.9 (19.4)		12.1 (11.8)		**<0.001**^**a**^		
mPPT	13.7 (1.4)		11.2 (3.1)		10.5 (3.2)		1.9 (3.3)		**<0.001**		
**Scale**	**CDR 0**		**CDR 0.5**		**CDR 1**		**CDR 2**		**CDR 3**		**p-value**
QPAR	39.1 (24.3)		21.8 (17.9)		16.7 (14.5)		10.7 (10.2)		8.2 (11.2)		**<0.001**^**b**^
mPPT	13.2 (1.5)		10.5 (2.8)		8.5 (3.1)		6.5 (3.6)		5.3 (2.4)		**<0.001**
**Scale**	**Control**		**MCI**		**AD**	**DLB**	**VaD**		**FTD**		**p-value**
QPAR	36.9 (25.2)		25.1 (21.4)		16.0 (15.2)	14.2 (13.9)	11.1 (8.2)		15.8 (18.7)		**<0.001**^**c**^
mPPT	13.1 (1.7)		10.9 (2.6)		8.6 (3.3)	7.6 (3.5)	7.0 (3.2)		10.0 (2.8)		**<0.001**

**KEY:** QPAR = Quick Physical Activity Rating; mPPT = Mini Physical Performance Test; CDR = Clinical Dementia Rating; MCI = Mild Cognitive Impairment; AD = Alzheimer’s Disease; DLB = Dementia with Lewy Bodies; VaD = Vascular Dementia; FTD = Frontotemporal Degeneration.

^a^Post-hoc analyses: Individuals 80 year and older are different from other age strata.

^b^Post-hoc analyses: CDR 0 different from all other CDR stages; CDR 0.5 not different from CDR 1; CDR 1, 2 and 3 are not different from each other.

^c^Post-hoc analyses: Controls different from MCI and all dementia etiologies; MCI different from all dementia etiologies; Dementia etiologies not different from each other.

## Discussion

The QPAR is a brief informant report of physical activity that corresponds to objective measures of physical performance and differentiates individuals with normal physical functionality from those individuals with impaired physical functionality confirming our hypothesis. The QPAR correlates with Gold Standard assessments of strength and sarcopenia (e.g., grip strength, SPSM), activities of daily living (e.g., FAQ), manual dexterity (i.e. Purdue pegboard), gait and mobility (e.g., TUG, computerized gait testing), frailty (e.g., Fried, CHSA ratings) and global physical performance (e.g., mPPT, SPPB). The QPAR as a measure of physical activity provided good discrimination between states of physical performance, functionality, falls risk, and frailty. The QPAR showed strong psychometric properties and excellent data quality, and worked equally well across different patient sexes, informant relationships, and in individuals with and without cognitive impairment and could be used as a brief informant-rated measure of physical activity.

Physical activity is a potentially modifiable risk factor for ADRD [19]. In clinical practice, however it can be difficult to gauge accurate histories of how much activity a person participates in, and in the case of older adults with cognitive impairment, histories may be unreliable. Conducting only a test of physical performance may give an inaccurate snapshot of what individuals do outside the clinical or research setting. Individuals with evidence of impaired cognitive function tend to perform poorly on physical tests [11, 57]. Even in older adults without evidence of cognitive impairment, faster performance on mobility tests is associated with better cognitive abilities [58]. This strong link between cognitive function and mobility has been interpreted as suggestive of an underlying aging process that accounts for declines across various systems including cognition and physical function [59] and may have shared risk factors [6]. A recent systematic review examined the relationship between physical frailty and cognition and found that 50% of studies reported a relationship with slowed gait, 40% with muscle weakness, 20% with exhaustion, and 10% with weight loss [60]. Moreover, changes in physical frailty and cognition are highly correlated, and the simultaneous decline in physical and cognitive function in late life likely reflects common underlying neuropathologies as evidenced by macroinfarcts, AD pathology, and nigral neuronal loss assessed in the brain at autopsy [61]. In a study of functional magnetic resonance imaging, changes in exercise over time were associated with frontal-subcortical network connectivity in older adults, independent of the presence of vascular disease, and were not related to neuropsychological changes. [62]. In addition to the effects of cognitive status on physical performance, advancing age also has measurable effects on physical activity, performance, and functionality. A recent study assessed the effects of an age simulation suit on gross motor, fine motor and cognitive performance in healthy young adults and found that the age simulation suit reduced all three performances as well as mood and perceived physical state [63].

There are several instruments available to measure physical activity in older adults. The most commonly used is the Physical Activity Scale for the Elderly (PASE) [23, 64, 65] developed in a community sample of older adults and correlated to health status and physiologic measures. The PASE contains 21 questions covering 12 activities over a 1-week period with the question about sitting activities not scored. Some questions ask about both frequency and duration, while others just ask about frequency. The PASE was positively correlated with grip strength, static balance, and leg strength, and negatively correlated with age, heart rate, perceived health status, and comorbidities [23]. The QPAR was correlated with these same measures. The PASE has been cross validated in a number of countries and cultures [66–68] and subsequently validated against an accelerometer [64]. The Patient-Reported Outcome Measurement Information System (PROMIS) Physical Function Scale [69] is a comprehensive scale with 124 items that comprise a wide range of activities using an algorithm to determine the patient’s physical performance score [70]. Other scales include the Community Health Activities Model Program for Seniors [71], General Practice Physical Activity Questionnaire [72], Modified Leisure Time Physical Activity Questionnaire [73], Stanford Brief Activity Survey [74], and Physical Activity and Sedentary Behavior Questionnaire (PASB) [73]. In a systematic review, most scales except for the PASE and PASB suffered from large measurement errors, low-quality evidence, and the lack of tests of reliability and construct validity [75]. Few of these scales were tested in individuals with cognitive impairment. The addition of the QPAR to the existing battery of tools could benefit researchers and clinicians looking for a measure of physical activity that has correspondence to measures of physical performance.

Advantages of measuring physical activity are multifold. Regular physical activity is essential to healthy aging and interventions to promote physical activity in older adults can have positive effects on health outcomes [4]. Recommendations for exercise and physical activity approach 150 minutes per week [76] but many older adults lead a sedentary life [4, 77], particularly with the development of cognitive impairment [11, 54]. The investigation of physical activity and its potential effect on outcomes requires that measurements of physical activity and the domains within the instrument reflect the multidimensionality of the construct. Questionnaires are commonly applied in intervention studies in older adults [75–78] and sufficient responsiveness of items is necessary to accurately measure changes of physical activity [75]. Recommendations for choosing a questionnaire to measure physical activity include [75–78]: sufficient content and construct validity, sufficient reliability, containing all relevant domains of physical activity (household, recreation, sport, transportation), capturing both frequency and duration of activity, having a recall period of at least 1 week, and the use of total time or time at different intensity levels for activities. The QPAR meets each of these criteria. While is it difficult to directly establish validity of a new instrument [54, 55], the evidence presented here supports that the interpretation of the QPAR is sound. The content validity was based on a review of the literature, the items had strong associations with hypothesized constructs of physical performance and physical functionality, known groups performed differently on the QPAR where expected, and the QPAR provided discrimination of physical functionality, falls risk, and frailty–hypothesized outcome consequences of low physical activity.

There are several limitations in this study. The QPAR collects information on reported physical activity and in this study is measured against objective measures of physical performance and functionality. Future studies could attempt to collect objective measures of physical activity in the home setting with the use of wearable sensors and smart devices and/or directly compare the QPAR to other existing measures of physical activity (e.g., PASE, PASB). The QPAR is reported by the informant covering a 4-week period and recall bias is possible. However, the QPAR reports of physical activity corresponded with objective measurements of physical performance suggesting that this was not a significant issue. The QPAR was validated in the context of an academic research setting where the prevalence of MCI and dementia are high, and the patients tend to be highly educated and predominantly White. Validation of the patient QPAR in other settings where dementia prevalence is lower (i.e. community samples) and the sample is more diverse is needed. As this is a cross-sectional study, the longitudinal properties of the QPAR still need to be elucidated. The QPAR was tested as an informant rating because of the cognitive impairment present in many of the patients and research participants. Future studies examining patient self-rating of physical activities are needed.

Strengths of this study include the use of a comprehensive evaluation that is part of standard of care with measurement of multiple Gold Standard measurement of strength, manual dexterity, mobility, physical performance, fitness, ADLs, health-related quality of life, frailty, and physical functionality to test our hypothesis that higher reported physical activity would correspond to better physical performance. Another advantage of the QPAR is its brevity (2–3 minutes) consisting of 10 questions to be printed on one piece of paper or viewed in a single screenshot to maximize its clinical and research utility. Unlike the PASE, the QPAR captures activities over a 4-week period, assigns weights to more intense activities, and captures both frequency and duration for all activities. This permits a calculation of dosage of activity that can be compared across different individuals. Unlike many of the available scales, the QPAR was studied in older adults with and without cognitive impairment.

The QPAR is an informant-rating outcome measure of physical activities in older adults and may serve as an effective clinical tool to determine the dose of physical activities that older adults are currently participating, predict physical functionality, and screen for frailty, sarcopenia, and falls risk. The QPAR may be useful for case-ascertainment in epidemiological studies and in busy primary care settings. The QPAR would not replace direct measurement of physical performance, but rather could be used as a complementary measure to provide a dosage of physical activity. As a measure of physical activity, the QPAR had good strength of association with these objective measures so it could provide an estimate of abilities prior to the formal assessment–it can be filled out at home or in the waiting room. In clinical practice, this could be helpful as the busy clinician might not have the time or capacity to perform a comprehensive physical performance evaluation (e.g., mPPT or TUG) on everyone but just those that have low reported levels of physical activity. Because of the wide range of activities and possible scores, the QPAR could help facilitate referrals to physical or occupational therapy providing the therapist with baseline activity, establish rehabilitation goals, assist in the assessment of improvement, and serve as an outcome measure [69]. Similarly, the QPAR could provide researchers with a baseline assessment of physical activity for an exercise intervention [79] and assist in determination of inclusion/exclusion criteria. The QPAR captured a range of physical activities and correlated with standardized scales of physical performance and physical functionality, providing a brief activity rating scale for use in clinical care and research.
